# Clinical Characteristics of Recurrent Nasopharyngeal Carcinoma in High-Incidence Area

**DOI:** 10.1100/2012/719754

**Published:** 2012-02-01

**Authors:** Jia-Xin Li, Tai-Xiang Lu, Ying Huang, Fei Han

**Affiliations:** ^1^State Key Laboratory of Oncology in South China, Sun Yat-Sen University, Guangzhou 510060, China; ^2^Department of Radiation Oncology, Sun Yat-Sen University Cancer Center, Guangzhou 510060, China

## Abstract

*Background*. To describe the clinical characteristics of the patients who suffered from relapse after conventional irradiation for nasopharyngeal carcinoma (NPC). *Methods*. Three hundred and fifty-one consecutive patients with first-time recurrent NPC between January 1999 and July 2005 were included. The patients' clinical data were reviewed, including recurrent interval time, symptoms, signs, imaging characteristics, pathologic features, and restaging. *Results*. The median interval of relapse was 26.0 months. The most common symptoms in symptomatic patients were nasal bloody discharge (37.9%) and headache (31.1%). Local recurrence alone accounted for 73.5%. Most patients were restaged as stage III (23.1%) and stage IV (51.1%). Subgroup analysis suggested a significantly higher proportion of the long-latent relapses originated from early primary. A series of postreirradiation complications were more frequent in patients with longer latency at reception. *Conclusions*. Most recurrent nasopharyngeal carcinoma is advanced disease. Patients with different recurrent interval time show different nature behavior.

## 1. Introduction

Nasopharyngeal carcinoma (NPC) is a common malignancy in Southern China. It poses a great challenge to radiation oncologists worldwide, because of its propensity for extensive local spread and its proximity to critical neural structures. Treatment results for nasopharyngeal carcinoma have improved in the past two decades. The improved treatment outcome has been attributed to earlier detection and institution of treatment, improved staging accuracy with the application of MRI, improved treatment planning with CT, improved dose distribution with intensity-modulated radiation therapy, and the use of chemotherapy though local failure is still a major hindrance to successful treatment. The 5-year cumulative rate of nasopharynx and cervical lymph node recurrence is between 12.0% and 22.0% [1–5]. Domestic and foreign studies have indicated that the clinical features of recurrent NPC may be different from primary NPC. Therefore, improving the recognition of the clinical features of recurrent NPC is of great significance to improving early diagnosis, early treatment, and the long-term survival rate for patients with recurrent NPC. This study summarized and retrospectively analyzed the relevant information of a large sample of patients with first-time recurrent NPC.

## 2. Materials and Methods

### 2.1. Patients

A retrospective review was conducted on case records of first-time recurrent NPC patients treated at the Cancer Center of Sun Yat-Sen University between January 1999 and July 2005. Patients who had complete response but developed recurrence at the primary site more than 4 months following completion of radical radiotherapy with or without chemotherapy were identified as having a recurrence. Most patients had undergone primary RT using a two-dimensional technique with two lateral opposing faciocervical fields in the first treatment phase, followed by two lateral opposing fields in the second phase to cover the nasopharynx, matched with a single anterior cervical field to cover the neck. The dose delivered to the nasopharynx was 66–70 Gy. Neoadjuvant, concurrent, or adjuvant chemotherapy, mostly cisplatin based, was given to 89 patients (25.4%) with advanced disease.

Patients were seen in followup 1 month postradiation and then every 3 months from 3 months through 3 years, and every 1 year thereafter. During every followup visit, disease status and treatment toxicity were assessed. Complete physical and fiberoptic nasopharyngoscopy or indirect nasopharyngeal speculum examinations were performed. Biochemistry profiles, chest radiography, abdominal ultrasonography, and contrast-enhanced CT or MRI of the nasopharynx and cervical region were routine elements of the assessment. Further investigations were arranged as indicated.

Total 351 patients were included. The majority were confirmed by biopsy, and all of them showed histological features similar to the primary tumor. Only those with recurrence at inaccessible sites such as the skull base, the cavernous sinus, and the intracranial area were diagnosed by radiological features. The criteria for diagnosis of recurrence without biopsy proof were based on progression of clinical symptoms and/or signs that are compatible of new image findings. Primary tumors were staged according to the 1992 Fuzhou staging system [6] as they were diagnosed, a system approved and recommended by the Chinese Association of Radiation Oncology in Fuzhou and widely used in mainland China since 1992. By the end of the 20th century, the UICC (International Union Against Cancer) and AJCC (American Joint Committee on Cancer) staging system had been well accepted in the high-incidence area. Therefore, the recurrent diseases were staged both according to the 1992 Fuzhou staging system and the 6th edition of the UICC and AJCC staging system [7]. A comparison between these two staging systems is summarized in Table 1.

For further study, the patients were divided into two groups according to the latent interval from commencement of primary treatment. Those who relapsed within 24 months were classified as group A, >24 months as group B. This breakpoint was chosen because (1) it was “landmark” most commonly used in reporting; (2) it was close to the median recurrence time (26 months) of this study; (3) it gave reasonable sample size in each group (169 patients in group A while 182 patients in group B).

### 2.2. Statistical Analysis

All analyses were performed in SPSS 16.0 for Windows. Variance analysis was used for processing the enumerated data, a *χ* ^2^ test was used for measurement of data, and a rank-sum test was used for ranked data. All statistical tests were two sided, and a *P* value of <0.05 was considered to indicate statistical significance.

## 3. Results

A total of 276 men (78.6%) and 75 women (21.4%) were included, with the male to female ratio of about 3.7 to 1. Patients' age ranged from 21.0 years to 73.0 years, with a median age of 46.0 years.

Of the 351 patients, 77.5% (272/351) were diagnosed on pathology as having recurrence: 0.7% (2/272) had World Health Organization (WHO) I (squamous cell carcinoma), 7.0% (19/272) had WHO II (nonkeratinizing carcinoma), and 92.3% (251/272) had WHO III (undifferentiated carcinoma). Some patients with only intracranial and skull base recurrence who were unable to have a biopsy performed were diagnosed by MRI, CT, and positron emission tomography (PET)-CT, at 12.3% (43/351), 8.0% (28/351), and 2.3% (8/351), respectively.

### 3.1. Recurrent Interval Time

**Figure 1:**
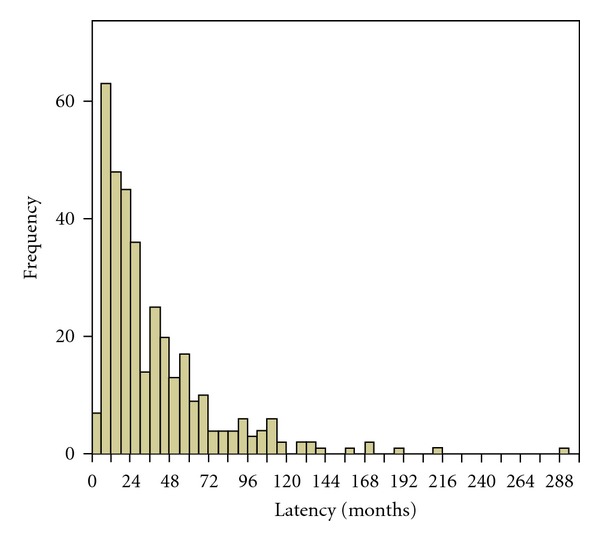
The relationship between latency and cases diagnosed as rNPC.

The duration from the end of the primary treatment to the diagnosis of first recurrence ranged from 4.0 months to 291.0 months, with a median recurrent interval time of 26.0 months, in which recurrent interval time less than or equal to 6 months accounted for 6.0% (21/351), 7 months to 12 months accounted for 17.4% (61/351), 13 months to 18 months accounted for 11.4% (40/351), and 19 months to 24 months accounted for 13.3% (47/351). The 2-year cumulative recurrence cases accounted for 48.1% (169/351). Recurrence from 25 months to 60 months accounted for 34.8% (122/351), from 61 months to 120 months accounted for 14.0% (49/351), and recurrent interval time more than 120 months accounted for 3.1% (11/351), as seen in Figure 1.

### 3.2. Clinical Presentation of Recurrence

Patients with recurrent disease with no obvious symptoms accounted for 10.3% (36/351), whose recurrence was found only at periodic reexamination. The most common symptoms in symptomatic patients were nasal bloody discharge [37.9% (133/351)] and headache [31.1% (109/351)], followed by tinnitus, neck mass, nasal obstruction, and hearing loss, which were 16.2% (57/351), 15.1% (53/351), 14.8% (52/351), and 11.7% (41/351), respectively. Other unusual symptoms included blurred vision, a sense that ears were plugged, facial edema, and epiphora; such symptoms did not exceed 3%. In addition, abducens dysfunction and facial numbness caused by cranial nerve damage were common symptoms in patients with recurrent NPC, accounting for 35.6% (125/351), of which the most common was for the damage of the abduction nerve and trigeminal nerve branch 2, accounting for 20.2% (71/351) and 19.1% (67/351), respectively, followed by the damage of trigeminal nerve branches 1 and 3, accounting for 10.0% (35/351) and 9.1% (32/351), respectively. The rate of other cranial nerve injuries was below 5%.

### 3.3. Patterns of Recurrence and Distant Metastasis

Recurrence in local alone was the most common [73.5% (258/351)], followed by the concurrent recurrence in both local and regional neck nodes [21.7% (76/351)]. Regional failure alone was rare [4.8% (17/351)]. Total cumulative rate of distant metastasis before the end of treatment for recurrence was 6.6% (23/351), of which metastasis occurred 43.5% (10/23) in bone, 21.7% (5/23) in distant lymph nodes, 13.0% (3/23) in the lungs, 4.3% (1/23) in the liver, 13.0% (3/23) in multiorgan sites, and 4.3% (1/23) in the right parotid gland and the right medial canthus.

### 3.4. Stages and Restages

For 27 patients first treated at other hospitals, the primary stages were unknown. 324 patients with primary nasopharyngeal carcinoma were retrospectively staged according to the 1992 Fuzhou staging system, of which 5.2% had stage I disease, 31.5% had stage II, 46.6% had stage III, and 16.7% had stage IVa disease.

Recurrent tumors were restaged as follows: stage I accounted for 7.4%, stage II accounted for 12.0%, stage III accounted for 27.4%, stage IVa accounted for 46.7%, and stage IVb accounted for 6.6%. As restaged according to the 6th edition of UICC and AJCC staging system, stage I accounted for 6.8% (24/351), stage IIa accounted for 1.7% (6/351), stage IIb accounted for 17.4% (61/351), stage III accounted for 23.1% (81/351), stage IVa accounted for 42.5% (149/351), stage IVb accounted for 2.0% (7/351), and stage IVc accounted for 6.6% (23/351). A correlation between the stages at initial diagnosis and at recurrence was shown in Table 2.

 The primary and recurrent stages according to the 1992 Fuzhou staging criteria were compared by Wilcoxon signed rank test. 52.5% (170/324) of patients' recurrent T-stages were more advanced than the primary T-stages, while 19.8% (64/324) of primary T-stages were more advanced than the recurrent T-stages (*P* < 0.001). 46.7% (151/324) of patients' recurrent N-stages were earlier than the primary N-stages, while 13.3% (43/324) of primary N-stages were earlier than the recurrent N-stages (*P* < 0.001). For the overall TNM stages, 53.4% (173/324) of patients' recurrent stages were more advanced than the primary stages, while 15.1% (49/324) primary stages were more advanced than the recurrent stages (*P* < 0.001).

### 3.5. Tumor Invasion

The common invasion sites of recurrent NPC revealed by imaging in the whole group included the skull base accounting for 54.4% (191/351), the prestyloid space for 43.3% (152/351), the carotid sheath area for 31.3% (110/351), the anterior group of cranial nerves for 28.2% (99/351), the cavernous sinus for 25.6% (90/351), the paranasal sinuses for 22.8% (80/351), the oropharynx for 18.5% (65/351), the nasal cavity for 17.9% (63/351), the intracranial invasion for 12.5% (44/351), the pterygomaxillary fossa for 11.4% (40/351), the posterior group of cranial nerves for 5.7% (20/351), the infratemporal fossa for 5.1% (18/351), the orbital apex for 5.1% (18/351), the soft palate for 3.1% (11/351), the cervical vertebrae for 2.3% (8/351), and the hypopharynx for 0.6% (2/351). Sites of primary and recurrent tumor invasion were compared by McNemar's test (Table 3). The results showed that the invasion rates of the oropharynx, the prestyloid space, and the carotid artery sheath by the primary tumor were significantly higher than those by the recurrence. Conversely, the invasion rates of the skull base, the paranasal sinuses, the cranial nerves, the cavernous sinus, the intracranial space, the pterygomaxillary fossa, the infratemporal fossa, the orbital apex, and the soft palate by recurrent tumors were significantly higher than those by the primary tumor.

### 3.6. Subgroups Compare

Group A and group B were compared in respect of the above (Table 4). The male to female ratio is higher in group A (5.26 : 1), while group B is 2.79 : 1 (*P* = 0.02). The median age first diagnosed as nasopharyngeal carcinoma is similar in two groups (*P* = 0.77). A significantly higher proportion of the long-latent relapses originated from early primary; the percentage of patients with T1 and T2 primary was 40.0% in group A, while 59.2% in group B; the percentage of patients with T3 and T4 primary was 60.0% in group A, while 40.8% in group B (*P* = 0.01). However, there was no difference in primary N-stage between two groups (*P* = 0.82).

The percentage of patients diagnosed as relapsed disease by pathology was lower in group A (70.4% versus 84.1%, *P* < 0.01). Nasal obstruction and hearing loss had significant difference between two groups among the symptoms while tumor relapsed. There were only 10.1% in group A with chief complaint of nasal obstruction, while 19.2% in group B (*P* = 0.02). Likewise, there were 6.5% in group A with chief complaint of hearing loss, while 16.5% in group B (*P* < 0.01). Higher incidence of postreirradiation complications was observed with longer latency. there were 2.4% in group A with neck fibrosis and induration, while 13.2% in group B (*P* < 0.01). The cumulative percentage of MRI scan evidenced temporal lobe encephalopathy in the two groups was 0% and 3.8%, respectively (*P* = 0.04). Though the differences of recurrent T-stage, N-stage, and the proportion of coexisting distant metastases were insignificant between two groups, the invasion rates of some structures associated with T4-stage were higher in group A. 9.6% in group A had the cavernous sinus invasion, while 3.4% in group B (*P* = 0.04). The percentage of intracranial invasion in the two groups was 16.6% and 8.8%, respectively (*P* = 0.04).

## 4. Discussion

Recurrence is one of the common failures after radiotherapy for nasopharyngeal carcinoma (NPC). A lot of reports focussed on the salvage treatment of recurrence, but few explored the characteristic of recurrent NPC specially. Our study was intended to shed more light on this aspect.

In our series, patients' median age is 46.0 years, which is consistent with that of other studies [8, 9]. The incidence of recurrent NPC was higher in men than in women. The male to female ratio was about 2.5 to 1 in some reports [8, 9], while 3.7 to 1 in ours, which is similar to the data reported by Teo et al. [10]. The mainly pathologic type of newly diagnosed patients in regions with high incidence was WHO III, so as our recurrent patients. A small part of the patients were not confirmed relapse disease by biopsy. The usual condition was the patients with tumor over deep submucosa, basilar skull, or intracranial area adjacent to the critical structures that might have technique difficulty or high morbidity probability for biopsy.

Similar to those of other reports [11, 12] with the median recurrent interval time of about two years, our data is 26.0 months. The frequency of recurrent cases reached its peak at 6 ~ 12 months after the end of the primary treatment then decreased gradually. Patients relapsed within 2 years accounted for nearly half of the total, which were considered at high risk for recurrence. Cases relapsed between 2 years and 5 years decreased by nearly one-third compared to those within 2 years, in which we should maintain sharp vigilance to the danger of recurrence. Those who relapsed between 5 years and 10 years accounted for only 14.0%, which indicated a low-risk period. Only very few patients were seen with relapse after 10 years (3.1%), which belonged to the stable period of cured NPC.

The painless enlargement of the upper cervical lymph node(s) was the most common presenting feature in about 40% to 75.8% of primary nasopharyngeal carcinoma [2, 3, 13], followed by nasal symptoms and aural problems. However, in this group, because isolated local failure relapse accounted for the majority of the recurrent patterns, neck mass was only found in 14.8% of chief complaints.s It should be noted that the incidence of headache is significantly high in our series, which was not mentioned in other reports [2, 3] of primary nasopharyngeal carcinoma expect for the study from HK [13]. This could be attributable to several factors: (1) the vast majority of recurrent patients were at late stages of disease, common with destruction at the base of the skull and/or intracranial invasion; (2) some patients had radiation encephalopathy after primary radiotherapy; (3) the radiation ulceration/necrosis complicated with infection made the situation worse. The incidence of cranial nerve injury in our series (35.3%) appears much higher than that in newly diagnosed patients (11.7% to 20.0%) [2, 3, 13]. The most frequently involved nerves were V, followed by VI, which is in accordance with a previous study [2]. Radiotherapy-related cranial nerve palsy may occur in patients with NPC after they receive conventional radiotherapy. But hypoglossal nerve palsy was found most frequently, followed by vagus nerve palsy and recurrent laryngeal nerve palsy [5, 14]. So the high incidence of cranial nerve injury in our series may be due to the extensive invasion of the recurrent disease, other than radiotherapy-related cranial nerve palsy.

Our data indicated that isolate local relapse is the most common recurrence pattern, which compares favorably with the results published in several contemporary series [5, 12, 15]. As to our knowledge, retreatment of locally recurrent NPC is associated with a high risk of complications [9]. What's more, patients with locoregional failure have an increased risk of distant metastasis [16]. Given the poor outcome and highly associated complications with retreatment, raising the local control rate is of great importance.

 In our patients, about 60% had advanced stages (stage III–IV) at initial diagnosis. By the time relapsed, the percentage had risen to over 80%. There have been a few studies which reported a detailed analysis of stages and restages of patients with recurrent nasopharyngeal carcinoma. Leung et al. [5] reported that of the patients with local recurrence who had early T classification at diagnosis, 70% had early T classification (rT1-2) at recurrence; of the patients with local recurrence who had advanced T classification (rT3-4) at diagnosis, 75% had advanced T classification at recurrence. Yu et al. [15] found that 50% of all local failures were advanced (rT3 and rT4). The recurrent T classification was the same as the initial T classification in 82% of the patients. But in the majority of our patients, recurrent T-stages were more advanced than the primary T-stages, while recurrent N-stages were earlier than the primary N-stages. For the overall TNM stages, more than half of the patients' recurrent stages were more advanced than the primary stages. All differences were statistically significant. Carefully analyzing the three studies, we found that the percentages of advanced stages at initial diagnosis in the other two series were much smaller than ours, which may be one of the reasons behind the susceptibility of the different results of stage and restage comparisons.

By comparing the invasion sites between primary and recurrent tumors, structures adjacent to the nasopharynx, such as the oropharynx, the prestyloid space, and the carotid artery sheath, the probability of invasion can be observed to be significantly lower in recurrent NPC. Conversely, regarding the structures far from the nasopharynx, such as the skull base, the paranasal sinuses, the cranial nerves, the cavernous sinus, the intracranial cavity, the pterygomaxillary fossa, the infratemporal fossa, the orbital apex, and the soft palate, the probability of invasion is significantly higher in recurrent disease than in primary disease. Areas neighboring the nasopharynx were in the high-dose area during the first treatment, while the areas far away from the nasopharynx received low or no dose exposure. So we speculate the possible explanation for this phenomena. The tumor cells of subclinical lesions in low-dose areas receive mostly sublethal damage and can be repaired, survive, continue to split, and lead to tumor recurrence? Another explanation is that the patients carry genes susceptible to NPC and had a tendency of recurrence under external factors, but the normal structure of the areas adjacent to the nasopharynx was destroyed by high-intensity rays at the first treatment, with the local blood supply reduced and unfavorable for tumor growth, so the tumor relocates and occurs far away from the nasopharynx. Furthermore, Nicholls et al. [17] suggested that at least a proportion of relapses were new growths rather than genuine recurrences, as 14% of cases showed extensive in situ changes. In such circumstance, it should come as no shock that the new tumor would arise from the area a little far away from the nasopharynx, where the blood supply is abundant.

The latency for nasopharyngeal carcinoma recurrence varies widely. For exploring if there is some difference in the clinical manifestation between recurrence with short latency and long latency, we made detailed analysis and comparison between the two groups (*⩽*2 years and >2 years). A lot of large studies [2, 18] showed a worse prognosis for male with nasopharyngeal carcinoma. In our series, the male to female ratio is significantly higher in group A. Lee et al. [11] reported that the prognosis of nasopharyngeal recurrence with long latency was significantly better. So the male tendency to relapse earlier should account for one of the explanations why they have worse prognosis. As most of the long survivors were patients with limited disease at presentation, it is not surprising that a higher proportion of long-latent relapse occurred in patients with early T-stages at the initial diagnosis. The percentage of patients diagnosed as relapsed disease by pathology was significantly lower in group A. Marx [19] reported that irradiation may cause hypoxia, hypovascularity, and hypocellularity and may impair normal collagen synthesis and cell production, which may lead to tissue breakdown. Then the lesion will be repaired by the proliferation of fibroblasts. Therefore, the pathological findings in a short time after irradiation are mainly necrotic tissue and fibrous tissue, which induce the rate of diagnosis by pathology to be lower in patients with short recurrence interval time.

Chen et al. [20] found that it was easy to lead to nasal synechia (the incidence rate was about 25.2%) when the dose of external irradiation is high or adding intracavitary irradiation or using the decongestant for long time. Ho et al. [21] reported that sensorineural hearing loss started early after irradiation in the patients with nasopharyngeal carcinoma. The acute adverse effect on bone conduction threshold recovered in 40% of ears at 2 years after irradiation. On long-term followup, the probability of sensorineural threshold deterioration increased. In other words, nasal synechia and hearing loss were common complications in patients after irradiation and became worse over time. Sometimes, it is difficult to distinguish whether they are complications or new symptoms. So nasal obstruction and hearing loss were seen more often in group B as recurrent symptoms. Likewise, there is no doubt that a series of postreirradiation complications such as neck fibrosis and temporal lobe encephalopathy were more frequent in patients with longer latency.

The differences of recurrent T-stage, N-stage, and the proportion of coexisting distant metastases were insignificant between two groups in our study, which conflicted with Lee's observation [11]. Lee et al. found that a significantly lower proportion of the long-latent relapse had tumors still confined within the nasopharynx (rT1) at detection of recurrence. As rT-stage was one of the most important prognostic factors, and the proportion of patients reirradiated was similar, they could not explain well why recurrence with long latency was associated with better prognosis. This problem will need to be further gone into. Perhaps we can expect a breakthrough at gene and protein level.

The current study describes the clinical characteristics of recurrent nasopharyngeal carcinoma. Patients relapsed within 2 years accounted for nearly half of the total. Isolated local relapse is the major recurrent pattern. Most patients had advanced disease at initial diagnosis, and the rate was even higher while recurrence. Subgroup analysis suggested that the male tents to relapse earlier. A higher proportion of long-latent relapse occurred in patients with early T-stages at initial diagnosis. A series of postreirradiation complications were more frequent in patients with longer latency at reception. More research is needed to improve the knowledge of recurrent nasopharyngeal carcinoma in hope to work out the prevention or radical cure of the disease.

## Figures and Tables

**Table 1:** Staging systems for nasopharyngeal carcinoma.

The sixth edition of the AJCC staging system	Fuzhou 1992 staging system of China
Tumor in nasopharynx (T)	Primary tumor (T)
T1: tumor confined to the nasopharynx	T1: Tumor confined to the nasopharynx
T2: tumor extends to soft tissues of oropharynx and/or nasal fossa T2a: without parapharyngeal extension T2b: with parapharyngeal extension	T2: Involvement of nasal fossa, oropharynx, soft palate prevertebral soft tissue, and parapharyngeal space extension before SO line*
T3: tumor invades bony structures and/or paranasal sinuses	T3: extension over SO line, involvement of anterior or posterior cranial nerves alone, skull base, pterygoid process zone, and pterygopalatine fossa
T4: tumor with intracranial extension and/or involvement of cranial nerves, infratemporal fossa, hypopharynx, orbit, or masticator space	T4: involvement of both anterior and posterior cranial nerves, paranasal sinus, cavernous sinus, orbit, infratemporal fossa, and direct invasion of first or second cervical vertebra
Regional lymph nodes (N)	Regional lymph nodes (N)
N0: no regional lymph node metastasis	N0: no enlarged lymph node
N1: unilateral metastasis in lymph node(s), ≤6 cm in greatest dimension, above the supraclavicular fossa	N1: the diameter of upper neck lymph node <4 cm, movable
N2: bilateral metastasis in lymph node(s), ≤6 cm in greatest dimension, above the supraclavicular fossa	N2: lower neck^§^ lymph node or the diameter between 4 and 7 cm
N3: metastasis in lymph node(s) N3a: >6 cm in greatest dimension N3b: extension to the supraclavicular fossa	N3: supraclavicular lymph node or the diameter >7 cm or fixed or skin infiltration
Distant metastasis (M) M0: no distant metastasis M1: distant metastasis	Distant metastasis (M) M0: absence of distant metastasis M1: presence distant metastasis
Stage grouping	Stage grouping
Stage I: T1 N0 M0	Stage I: T1 N0 M0
Stage IIA: T2a N0 M0	Stage II: T2 N0-1 M0
Stage IIB: T1-2 N1 M0; T2b N0 M0; T1-2	Stage III: T3 N0-2 M0; T1-3 N2 M0
N1 M0	Stage IVA: T4 any N, M0
Stage III: T1-3 N2 M0; T3 N0-1 M0	Stage IVB: any T, any N, M1
Stage IVA: T4 N0-2 M0; any T N3, M0	
Stage IVB: any T N3 M0	
Stage IVC: any T, any N M1	

*The line connected from the styloid process to the midpoint on posterior edge of the great occipital foramen.

^§^The border between upper and lower neck is the lower margin of the cricoid cartilage.

**Table 2:** Stage and restage according to the 1992 Fuzhou staging system.

Initial stage	No. of patients by recurrent stage					Total
	I	II	III	IVa	IVb	
I	2	4	4	7	0	17
II	11	15	30	40	6	102
III	8	18	46	67	12	151
IVa	3	2	7	39	3	54

Total	24	39	87	153	21	324

**Table 3:** Comparison of tumor invasion in patients with pNPC* and rNPC.

Tumor invasion	No invasion in rNPC (case)	Invasion in rNPC (case)	*P* value
Oropharynx			<0.01
No invasion in pNPC (case)	157	31	
Invasion in pNPC (case)	66	20	
Nasal cavity			0.81
No invasion in pNPC (case)	189	37	
Invasion in pNPC (case)	34	14	
Soft palate			<0.01
No invasion in pNPC (case)	265	9	
Invasion in pNPC (case)	0	0	
Prestyloid space			<0.01
No invasion in pNPC (case)	52	32	
Invasion in pNPC (case)	105	85	
Carotid sheath area			<0.01
No invasion in pNPC (case)	114	41	
Invasion in pNPC (case)	80	39	
Pterygopalatine fossa			<0.01
No invasion in pNPC (case)	230	30	
Invasion in pNPC (case)	10	4	
Skull base			<0.01
No invasion in pNPC (case)	114	102	
Invasion in pNPC (case)	16	42	
Anterior group of cranial nerve			<0.01
No invasion in pNPC (case)	186	65	
Invasion in pNPC (case)	10	13	
Posterior group of cranial nerve			<0.01
No invasion in pNPC (case)	259	13	
Invasion in pNPC (case)	2	0	
Paranasal sinuses			<0.01
No invasion in pNPC (case)	194	54	
Invasion in pNPC (case)	15	11	
Infratemporal fossa			<0.01
No invasion in pNPC (case)	256	15	
Invasion in pNPC (case)	3	0	
Orbital apex			0.03
No invasion in pNPC (case)	255	14	
Invasion in pNPC (case)	4	1	
Cavernous sinus			<0.01
No invasion in pNPC (case)	197	60	
Invasion in pNPC (case)	8	9	
Cervical vertebrae			0.06
No invasion in pNPC (case)	269	5	
Invasion in pNPC (case)	0	0	
Intracranial			<0.01
No invasion in pNPC (case)	238	33	
Invasion in pNPC (case)	1	2	
Laryngopharynx			0.50
No invasion in pNPC (case)	272	2	
Invasion in pNPC (case)	0	0	

*Primary nasopharyngeal carcinoma (total 274 patients had clear radiographic image data).

**Table 4:** Changes in clinical features with increasing latency.

Features	Group A (*⩽*2 y)	Group B (*＞*2 y)	*P* value
Sex			0.02
Male	142	134	
Female	27	48	
Age	43.0	42.5	0.77
T-stage*			0.01
T1	9	30	
T2	53	70	
T3	62	51	
T4	31	18	
N-stage*			0.82
Diagnosis			<0.01
Pathology	119	153	
CT	16	12	
MRI	31	12	
PET-CT	3	5	
rT-stage*			0.43
rT0	10	7	
rT1	14	18	
rT2	18	24	
rT3	42	56	
rT4	85	77	
rN-stage*			0.38
rM-stage*			0.52

*Staged by the 1992 Fuzhou staging system (total 324 patients had clear pretreatment stages).
